# Update and audit of the St George’s classification algorithm of primary lymphatic anomalies: a clinical and molecular approach to diagnosis

**DOI:** 10.1136/jmedgenet-2019-106084

**Published:** 2020-05-14

**Authors:** Kristiana Gordon, Ruth Varney, Vaughan Keeley, Katie Riches, Steve Jeffery, Malou Van Zanten, Peter Mortimer, Pia Ostergaard, Sahar Mansour

**Affiliations:** 1 Molecular and Clinical Sciences Research Institute, St George’s University of London, London, UK; 2 Dermatology & Lymphovascular Medicine, St George's Universities NHS Foundation trust, London, UK; 3 Lymphedema Clinic, Derby Hospitals NHS Foundation Trust, Derby, UK; 4 Molecular and Clinical Sciences Research Institute, St George's, University of London, London, UK; 5 SW Thames Regional Genetics Service, St George’s Hospital, London, UK

**Keywords:** central conducting lymphatic anomaly (CCLA), lymphatic, generalised lymphatic anomalies (GLA), primary lymphoedema

## Abstract

Primary lymphatic anomalies may present in a myriad of ways and are highly heterogenous. Careful consideration of the presentation can lead to an accurate clinical and/or molecular diagnosis which will assist with management. The most common presentation is lymphoedema, swelling resulting from failure of the peripheral lymphatic system. However, there may be internal lymphatic dysfunction, for example, chylous reflux, or lymphatic malformations, including the thorax or abdomen. A number of causal germline or postzygotic gene mutations have been discovered. Some through careful phenotyping and categorisation of the patients based on the St George’s classification pathway/algorithm. The St George’s classification algorithm is aimed at providing an accurate diagnosis for patients with lymphoedema based on age of onset, areas affected by swelling and associated clinical features. This has enabled the identification of new causative genes. This update brings the classification of primary lymphatic disorders in line with the International Society for the Study of Vascular Anomalies 2018 classification for vascular anomalies. The St George’s algorithm considers combined vascular malformations and primary lymphatic anomalies. It divides the types of primary lymphatic anomalies into lymphatic malformations and primary lymphoedema. It further divides the primary lymphoedema into syndromic, generalised lymphatic dysplasia with internal/systemic involvement, congenital-onset lymphoedema and late-onset lymphoedema. An audit and update of the algorithm has revealed where new genes have been discovered and that a molecular diagnosis was possible in 26% of all patients overall and 41% of those tested.

## Introduction

The lymphatic system is a network of vessels important for whole body fluid homeostasis, lipid absorption and immune cell trafficking.^1 2^ Lymphoedema is caused by lymphatic dysfunction, which leads to a build-up of interstitial fluid within the tissues. This manifests with swelling of the extremities, usually of the legs but may involve other regions or segments of the body such as the upper limbs, face, trunk or genital area. There is an increased risk of infection due to disturbances in immune cell trafficking within the segment of compromised lymph drainage.^3^ Lymphatic dysfunction within the thorax and abdomen, here referred to as systemic/internal involvement (but can be referred to as visceral or central involvement), may present with pleural or pericardial effusions or ascites, any of which may be chylous, as well as intestinal or pulmonary lymphangiectasia, protein losing enteropathy or chylous reflux.

The International Society for the Study of Vascular Anomalies (ISSVA) updated their classification for vascular anomalies in 2018.^4^ The vascular malformations are subgrouped into ‘combined’, which include more than one type of vessel, ‘simple’ (only involving one vessel type), and those ‘associated with other anomalies’.

Lymphoedema due to a presumed genetic developmental fault in the structure or function of lymph conducting pathways is called primary lymphoedema.^5^ Some developmental faults can lead to overt structural defects of the lymph conducting pathways and are called lymphatic malformations. Such malformations if interfering with lymph drainage cause lymphoedema (truncal malformations) but some lymphatic malformations remain as isolated anomalies with no connection to main lymph drainage pathways and do not cause lymphoedema (non-truncal malformations).^6^ A primary lymphatic anomaly is an umbrella term referring to all lymphatic abnormalities arising from a developmental fault.

For a long time, the diagnosis of primary lymphoedema was based largely on the age of presentation of the swelling, congenital, pubertal and late onset, with limited differentiation between the phenotypes. The discovery of the first causal gene, vascular endothelial growth factor receptor 3 for Milroy disease, indicated that a molecular diagnosis was possible.^7^ The first St George’s classification algorithm of primary lymphoedema and other primary lymphatic disorders was an attempt to guide a clearer categorisation of phenotypes and enable the discovery of further causal genes.^8^ Age of onset remained a key criterion, but the sites affected and associated features, for example, dysmorphology, distichiasis (aberrant eyelashes), varicose veins, vascular malformations and limb overgrowth were also considered, as was internal or systemic involvement, for example, fetal hydrops, intestinal lymphangiectasia, pleural and pericardial effusions and chylous reflux. A family history of lymphoedema with determination of the mode of inheritance was considered useful.

More rigorous phenotyping facilitated the identification of subgroups of patients with the same broad category of primary lymphatic anomaly. These cohorts were then used for molecular studies to identify more causal genes. Once the genotype was known then crosschecking of the clinical characteristics, natural history and inheritance patterns was possible and an accurate phenotype defined. Investigations such as lymphoscintigraphy helped to refine the phenotype further and give insight into the mechanisms for the development of the lymphatic disorder. A first update of the classification was published in 2013.^9^

The St George’s classification algorithm is intended to help clinicians categorise their patients and guide testing towards, where possible, a molecular diagnosis. This algorithm is criteria matching, that is, using certain key findings for classification through a multistep process of history taking, examination findings, mutation testing, etc. The next step using the information gathered is to advise on natural history, prognosis and risk (including genetic counselling) and to guide management. While a molecular diagnosis should provide the most specific and accurate diagnosis, it can be seen particularly with the postzygotic mosaic disorders that one genotype can be clinically very heterogenous so there will probably always be a place for good clinical phenotyping supported by investigation to guide management.

Here, we present a second update of the St George’s classification algorithm to include newly discovered genes and to bring it in-line with the 2018 ISSVA classification for vascular anomalies.^4^ The results of an audit, the purpose of which was to determine how well the algorithm was performing as a diagnostic aid to classify patients with primary lymphatic anomalies and guide molecular testing are also presented.

## Methods

### St George’s classification algorithm of primary lymphatic anomalies

**Figure 1:**
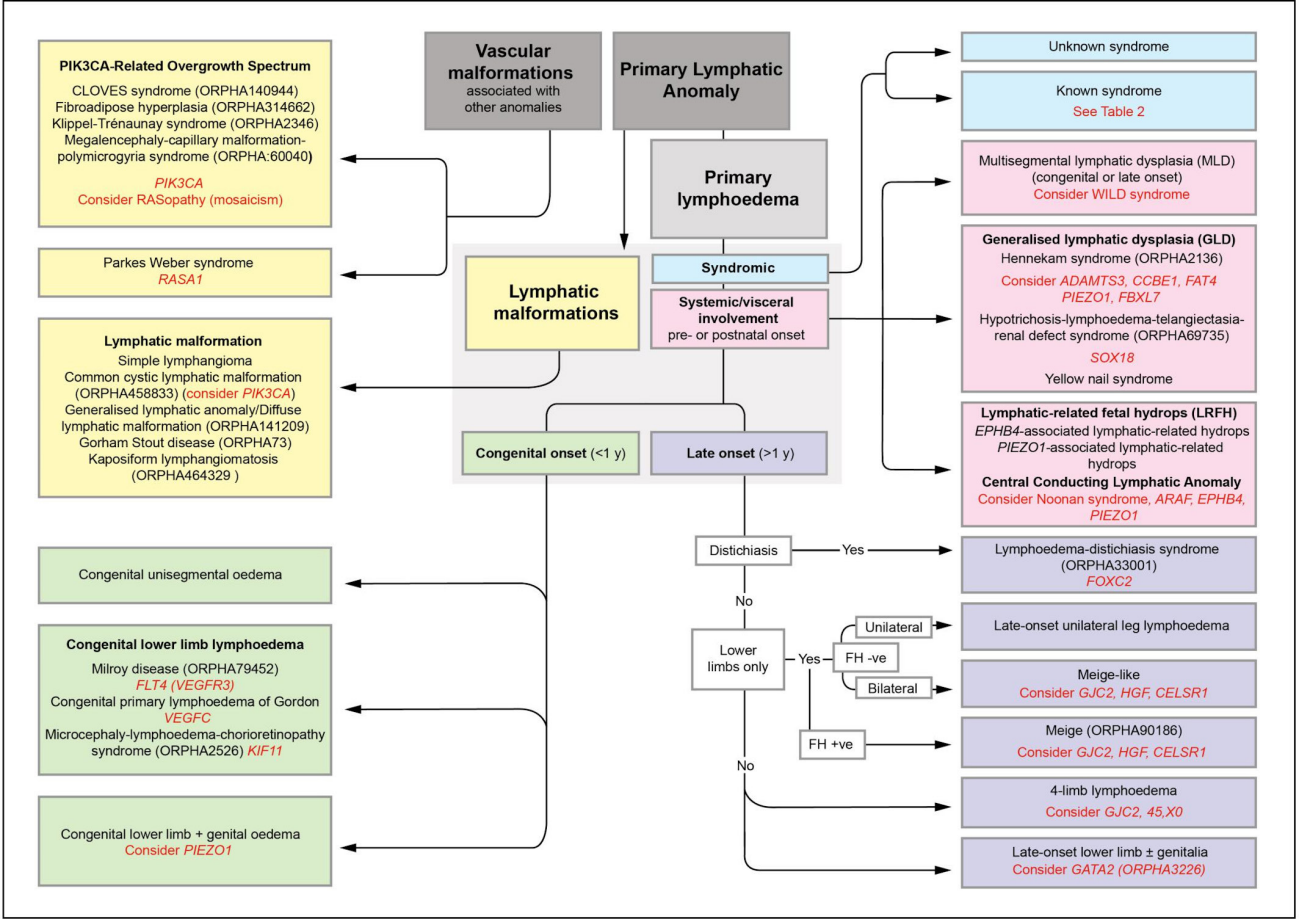
St George’s classification algorithm for primary lymphatic anomalies. The five main groupings (colour coded) with their various clinical subtypes of disease. Primary lymphoedema is the major clinical feature in the green, pink and purple sections. Text in red indicates the suggested genetic test and/or differential diagnosis for the subgroup, however, the indicated genes do not explain the cause of disease in all patients in each grouping. For example, only 70% of patients with Milroy disease are explained by mutations in *FLT4/VEGFR3*.^33^ FH, family history; +ve, positive; −ve, negative. (Image shared by St George’s Lymphovascular Research Group under the CC BY-SA 4.0 International licence on Wikimedia Commons).

The St George’s classification algorithm was updated (figure 1) and then applied, retrospectively, to all patients presenting to the national multidisciplinary ‘Primary and Paediatric Lymphoedema’ Clinic held at St George’s Hospital over a 1-year period. Careful phenotyping was undertaken both on clinical grounds and after selective investigations, for example, lymphoscintigraphy. Where possible and appropriate, targeted genetic testing was performed (this was prior to the introduction of a lymphoedema gene panel in our unit) for some of the genes listed in table 1.

**Table 1:** An overview of genetic disorders with primary lymphoedema as a frequent and dominant feature, categorised by inheritance and age of onset

		**Disorder**	**ORPHANET**	**OMIM**	**GENE**
**Autosomal dominant**	**Late onset (>1 year**)	Lymphoedema distichiasis syndrome (LDS)	33 001	153 400	*FOXC2*
		Late onset 4-limb lymphoedema		613 480	*GJC2*
		Emberger syndrome / Primary lymphoedema with myelodysplasia	3226	614 038	*GATA2*
		Late-onset hereditary lymphoedema			*CELSR1*
		Meige disease	90 186	153 200	*?*
	**Congenital (<1 year**)	Milroy disease	79 452	153 100	*VEGFR3/FLT4*
		Congenital primary lymphoedema of Gordon		615 907	*VEGFC*
		Microcephaly-chorioretinopathy-lymphoedema syndrome	2526	152 950	*KIF11*
		Capillary malformation-arteriovenous malformation (CMAVM)		608354 618 196	*RASA1EPHB4*
		Autosomal dominant lymphatic-related foetal hydrops (LRFH)		617 300	*EPHB4*
		Hypotrichosis-lymphoedema-telangiectasia-renal defect syndrome (HLTRS)	69 735	137 940	*SOX18*
**Autosomal recessive**		Hypotrichosis-lymphoedema-telangiectasia syndrome (HLTS)	69 735	607 823	*SOX18*
		Hennekam-lymphangiectasia-lymphoedema syndrome Type 1, 2 and 3	2136	235510 616006 618 154	*CCBE1FAT4ADAMTS3*
		Generalised lymphatic dysplasia of Fotiou		616 843	*PIEZO1*
**Mosaicism**		PIK3CA-related overgrowth spectrum (PROS)		171 834	*PIK3CA*
		Mosaic RASopathies			e.g. *KRAS/NRAS/HRAS/MAP2K1*

Their position in the classification pathway is indicated by the same colour coding as used in figure 1. For each disorder, the causal gene, Orphanet and OMIM IDs are given where known.

Within the St George’s classification algorithm (figure 1), there are five main categories of primary lymphatic anomalies. These are presented in the form of colour-coded sections with the individual subtypes (including genotypes) within the categories. For definitions of some of the terms used, see Glossary of Terms (see online supplementary section).

10.1136/jmedgenet-2019-106084.supp1 Supplementary data

First, the yellow section includes the ‘vascular malformations associated with other anomalies’ and the ‘lymphatic malformations’ (as defined in the ‘Introduction’ section).

Second, the patient is assessed for syndromes that have lymphoedema as a non-dominant feature (blue section), for example, the patient is dysmorphic with learning difficulties and possibly has other abnormalities.

Then if not obviously syndromic, and the lymphatic problems are the dominant feature, further assessment and investigations for systemic/internal lymphatic dysfunction or central conducting anomalies (eg, chylothoraces, chylopericardial effusions, ascites or protein losing enteropathy) are undertaken (pink section). These include a careful medical history asking specifically about prenatal history (eg, hydrothoraces, fetal hydrops), chronic diarrhoea, abdominal bloating or discomfort with fatty foods, weight loss or faltering growth (in a child) or shortness of breath on exertion. Blood investigations (including serum albumin, immunoglobulins, lymphocyte subsets, faecal levels of calprotectin or alpha-1-antitrysin), echocardiograms and chest radiographs are helpful if central lymphatic dysfunction is suspected.

Where none of the above features is present, then the age of onset is used to determine the grouping; the green section deals with congenital-onset primary lymphoedema (includes syndromes where lymphoedema is the dominant clinical problem, and which is present at birth or develops within the first year of life but is not associated with systemic/internal lymphatic dysfunction). The purple section addresses late-onset primary lymphoedema (ie, lymphoedema that is the dominant clinical problem, and which develops after the first year of life but is not associated with systemic/internal lymphatic dysfunction). It was decided not to differentiate between pubertal onset (praecox) and later onset in life (tarda) when it was discovered that one genotype such as *FOXC2* can cause both.

It is important to note that the specific diagnosis may be difficult in a neonate presenting with isolated congenital primary lymphoedema. A baby born with lymphoedema may later present with developmental delay, systemic involvement, progressive segmental overgrowth or a vascular malformation, which could suggest a diagnosis in one of the other categories. It should also be emphasised that each colour-coded section is not exclusive. Some somatic overgrowth anomalies may possess significant internal involvement. Also, lymphoedema distichiasis syndrome is allocated to the purple late-onset lymphoedema section because the dominant feature is the late-onset lymphoedema not the associated features, which make it a syndrome. The blue ‘syndromic’ section refers to conditions with a collection of features where lymphoedema is not the main characteristic. The algorithm is intended to guide a clinical diagnosis and target gene testing.

### Genetic methodology

For the purposes of the audit, targeted genetic testing of FOXC2, VEGFR3, CCBE1, SOX18, RASopathy genes and PIK3CA was performed by Sanger sequencing of DNA extracted from lymphocytes or skin fibroblasts in patients in whom a specific genetic diagnosis was suspected. This was before the introduction of a lymphoedema gene panel. Some patients, who were either negative for the targeted genes or did not fit the relevant phenotypes of those genes, were included in Whole Exome Sequencing (WES) cohorts after classification, which then led to the identification of new disease genes such as EPHB4, GATA2, PIEZO1, GJC2 and FAT4.

### Retrospective audit of the St George’s Clinic for 2016

A 12-month retrospective audit for the year 2016 (1 January 2016–31 December 2016) was performed. The aim of the audit was to look at the proportion of patients in each category of the classification algorithm and to look at the success of making a molecular diagnosis through use of the algorithm. The audit criteria required the patients to be seen in our specialist clinic, at any age, with a diagnosis of a primary lymphatic anomaly with data collected from medical records and laboratory results.

## Results

### Results of the retrospective audit

Over a 12-month period in 2016, 227 patients were seen (age range 2 weeks to 70 years), 25.6% (n=58/227) of which were new patients. Over one-third (38%) of patients seen in the clinic had a family history of primary lymphoedema.

Few patients had received genetic testing prior to referral to the clinic. Targeted genetic testing was completed in 63% (n=143) of the patients seen. At that time, a lymphoedema gene panel was not available, patients were only tested if the clinician felt there was a reasonable chance of finding a molecular cause, that is, testing was targeted.

Of those tested, the underlying genetic cause was identified in 41% (n=59/143). Overall, a molecular diagnosis was made in 26% (59/227) of all the patients seen in 2016.

### Vascular malformations with associated anomalies and lymphatic malformations (yellow)

**Figure 2:**
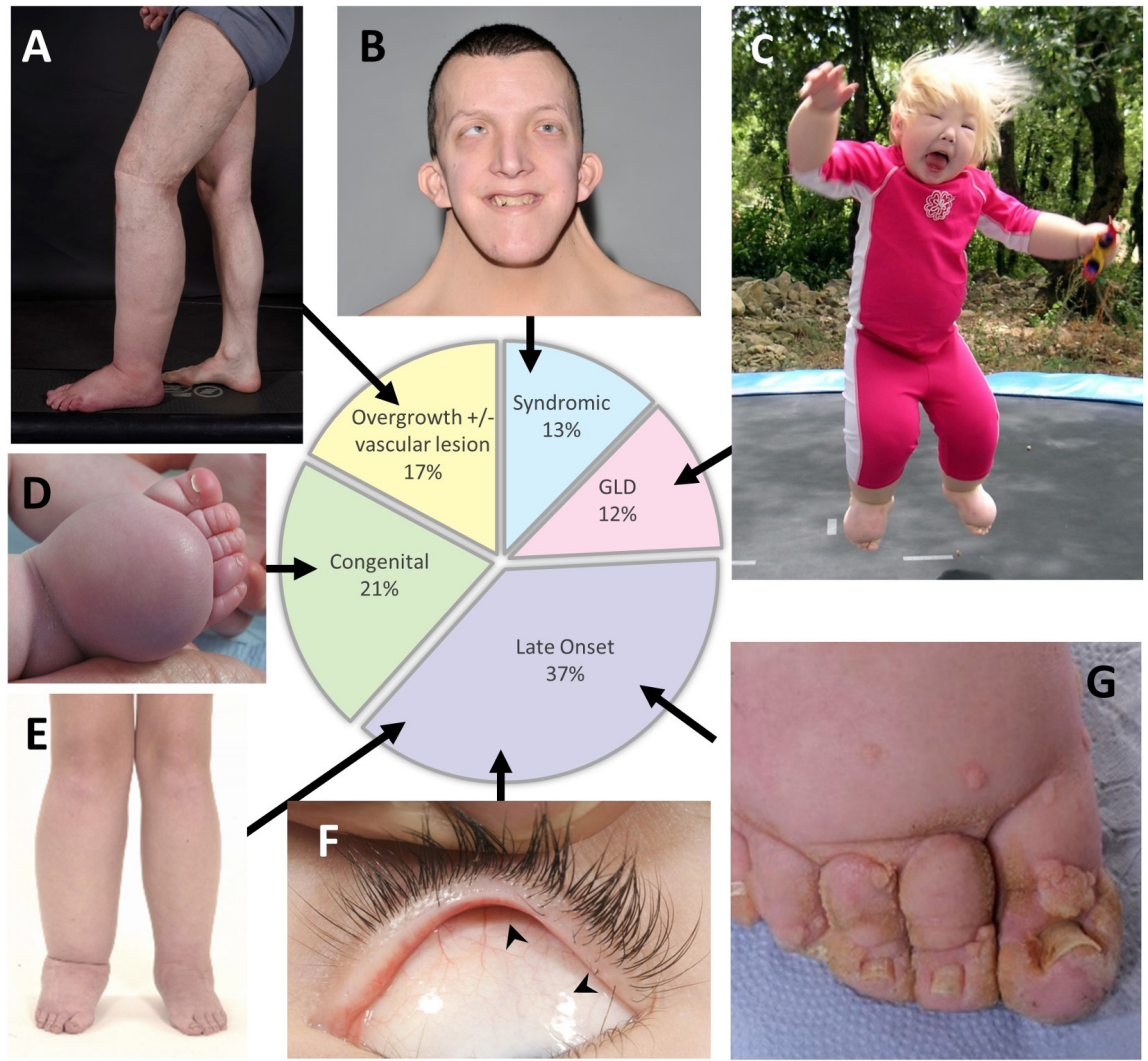
A graphic representation of the 227 audited patients seen in clinic in 2016 and their distribution across the five categories from figure 1 (pie chart). (A–G) Images show features of each category. (A) Patients with postzygotic mutations often present with asymmetrical swelling and segmental overgrowth as this patient, who is mosaic for a mutation in *KRAS*. (B) Webbed neck in Noonan syndrome. (C) In rare cases, swellings can be widespread affecting all segments of the body such as in this child with biallelic *CCBE1* mutations. (D) In milder forms, often just the dorsum of the foot is affected as in this baby with a *VEGFR3* mutation. (E, F) Lower limb swelling and distichiasis (arrowheads in F) in a patient with a *FOXC2* mutation. (G) Lymphoedema is a major cause of skin disease and affected patients suffer from severe and recurrent episodes of cutaneous infection, especially HPV-associated warts as seen in patients with *GATA2* mutations. GLD, generalised lymphatic dysplasia.

This group presents with malformations in the structure and organisation of blood and lymphatic vessels with a patchy, segmental distribution. Lymphoedema may develop in combination with vascular malformations and segmental overgrowth (or occasionally, undergrowth) of tissues within the swollen limb, for example, muscle, skeletal or adipose tissues (figure 2A). The combination of lymphatic and vascular malformations in this group reflects the mutual embryological origins of the two vascular systems.

These conditions are usually due to postzygotic mutations, for example, *PIK3CA-*related overgrowth spectrum (PROS)). Exceptions to this are capillary malformation-arteriovenous malformation (MIM 608354) such as Parkes-Weber syndrome, which may be caused by heterozygous, germline mutations in *RASA1*.^10^

Of the 227 patients seen in 2016, 17% (n=39) had lymphoedema associated with vascular malformations and/or segmental overgrowth (or undergrowth) (figure 2, pie chart) in comparison with 15% in 2010.^8^ It has been shown that postzygotic, gain of function mutations in *PIK3CA* may be responsible for many of the mosaic segmental overgrowth spectrum disorders.^11^ Postzygotic mutations are rarely identified in blood samples and therefore require a skin biopsy of the affected region. In the 2016 cohort, only 10 patients (26%) provided skin biopsies for genetic analysis, producing just one molecular diagnosis. More research in this field is required to identify the genetic basis for some of the conditions in this category. However, since the last revision, we have gained a much better understanding of the classification of some of these postzygotic mosaic conditions, therefore a brief review of the latest developments in this area is given in the online supplementary section.

### Syndromic lymphoedema (blue)

Syndromes associated with primary lymphatic anomalies are listed in table 2 and include chromosomal abnormalities, single gene disorders and imprinting disorders. Patients attending the clinic with syndromic primary lymphoedema made up 13% (n=29) (figure 2, pie chart), similar to the 15% reported by Connell *et al*.^8^ Nearly three-quarters (72%, n=21) of this cohort had a molecular or chromosomal diagnosis. The most frequently seen syndromes were Noonan syndrome (n=8) (figure 2B), Turner syndrome (n=4) and Phelan McDermid syndrome (n=3).

**Table 2:** An overview of ‘Known Syndromes’ with primary lymphoedema as a non-dominant association as referred to in the St George’s classification algorithm (figure 1, blue section)

Syndromes caused by chromosomal abnormality		OMIM	Chromosome
Phelan McDermid syndrome		606 232	22q terminal deletion or ring chromosome 22
Prader Willi syndrome		176 270	15q11 microdeletion or maternal UPD 15
Thrombocytopenia with absent radius		274 000	1q21.1 microdeletion and *RBM8A*
Turner syndrome			45, X0
Velocardiofacial syndrome		192 430	22q11 microdeletion
**Syndromes caused by single gene fault**		**OMIM**	**Gene(s**)
Autosomal dominant	Noonan/Cardiofaciocutaneous syndrome	163 950	*PTPN11, RIT1, RAF1, SOS1, KRAS, BRAF, MAP2K1, MAP2K2* plus others
	CHARGE syndrome	214 800	*CDH7*
	Microcephaly-chorioretinopathy-lymphoedema-intellectual disability	152 950	*KIF11*
	Oculo-dento-digital syndrome	164 200	*GJA1*
	Hypotrichosis-lymphoedema-telangiectasia-renal-defect syndrome	137 940	*SOX18*
	Ruvalcaba syndrome	180 870	*PTEN*
	Costello syndrome	218 040	*HRAS*
	Sotos syndrome	117 550	*NSD1*
	Tuberous sclerosis	191 100	*TSC1, TSC2*
Autosomal recessive	Carbohydrate-deficient glycoprotein types 1a, 1b and 1 hour	212 065, 602 579, 608 104	*PMM2, PM1, ALG8*
	Choanal atresia-lymphoedema	613 611	*PTPN14*
	Cholestasis-lymphoedema syndrome (Aagenaes syndrome)	214 900	
	Hennekam-lymphangiectasia-lymphoedema syndrome type 1, 2 and 3	235 510 to 616 006	*CCBE1, FAT4, ADAMTS3*
	Hypotrichosis-lymphoedema-telangiectasia syndrome	607 823	*SOX18*
X linked	Ectodermal dysplasia, anhidrotic, immunodeficiency, osteopetrosis and lymphoedema syndrome	300 301	*IKBKG (NEMO*)
	Fabry disease	301 500	*GLA*
Somatic	CLOVES syndrome Macrocephaly capillary malformation	602 501	*PIK3CA*
**Syndromes with no known cause**		**OMIM**	
Irons-Bianchi syndrome		601 927	
Mucke syndrome		247 440	
Progressive encephalopathy, hypsarrhythmia, optic atrophy		260 565	
Yellow nail syndrome		153 300	

The syndromes are categorised by mode of inheritance. The causal genes or structural variants and OMIM number are indicated where known.

### Lymphoedema with prenatal or postnatal systemic involvement (pink)

In some conditions, lymphoedema may be associated with internal (systemic or visceral) disturbances of the lymphatic system within thorax or abdomen, for example, fetal hydrops, intestinal lymphangiectasia (presenting as protein-losing enteropathy), pulmonary lymphangiectasia or with pericardial and/or pleural effusions (often chylous), or chylous reflux (often into the genitalia). Broadly, there are two types of lymphoedema with systemic involvement: (A) ‘widespread’ swelling affecting all segments of the body (figure 2C), such as that seen in generalised lymphatic dysplasia (GLD). Due to faulty development, the structural or functional abnormality of the lymphatic system is affecting the whole body. One type is Hennekam-lymphangiectasia-lymphoedema syndrome^12^; (B) ‘patchy’ areas of swelling, for example, left arm and right leg, which have been named ‘multisegmental lymphatic dysplasia’ (MLD) (figure 1).

Prenatally, these conditions may present with pleural effusions (hydrothoraces), or as non-immune fetal hydrops (the accumulation of fluid in at least two compartments of a fetus such as the abdominal cavity, pleura or subcutaneous oedema). Fifteen per cent of non-immune cases of hydrops are the result of lymphatic disorders, and approximately 20% are idiopathic, some of which may be due to, as yet, unidentified lymphatic abnormalities.^13^

In our audit, this cohort accounted for 12% (n=27) of patients (figure 2, pie chart), slightly higher than the 8% reported in 2010.^8^ Molecular testing was carried out in 17 patients. Nine of those tested had GLD, and pathogenic variants were identified in seven (78%). Five had biallelic variants in the *PIEZO1* gene and one each with biallelic variants in *FAT4* and *SOX18*. Interestingly, two of the families described by Connell *et al*, cases 3 and 4, have subsequently been found to be caused by biallelic variants in the *PIEZO1* gene.^8 14^

None of the eight patients, who presented with ‘patchy’ distribution of lymphoedema (MLD), had an identifiable molecular diagnosis. It is suspected that these patients could have a postzygotic mosaic mutation or WILD syndrome.^15^

Since the last revision of the St George’s classification algorithm was published,^9^ five new causal genes associated with GLD and/or non-immune fetal hydrops have been identified: *ADAMTS3*,^16^ *EPHB4*,^17^ *FAT4*,^18^ *FBXL7* 19 and *PIEZO1* 14 20 and are reviewed in the online supplementary section.

### Congenital onset lymphoedema (green)

In this category, congenital onset is defined as lymphoedema that is present at birth or develops within the first year of life. Bilateral lower limb swelling is the most frequent presentation (figure 2D), but the swelling may be unilateral and/or involve the arms, genitalia and/or face, depending on the underlying cause. There are a number of different genetic disorders presenting with congenital lymphoedema (table 1). Milroy disease (ORPHA79452; OMIM 153100) is the most common form, occurring as a result of pathogenic variants in *FLT4/VEGFR3*.^21 22^ The mutation may occur de novo, so a family history is not essential for this diagnosis. The lymphoedema is always confined to the lower limbs but may be unilateral, and may (rarely) involve the genitalia. Approximately 10% of mutation carriers do not have lymphoedema. Fetuses with Milroy disease may present antenatally with pedal oedema in the third trimester, and, in a few cases, with bilateral hydrothoraces, which resolve before birth.

Pathogenic variants in *VEGFC*, the ligand for VEGFR3, have also been identified in association with congenital primary lymphoedema of Gordon (OMIM 615907), also affecting the lower limbs.^23–26^

The congenital category represents 21% (n=47) of the patients seen in 2016 (figure 2, pie chart) compared with 24% in 2010.^8^ A pathogenic variant was identified in 19 of the 47 (40%) patients genetically tested in this category. The majority (n=18) had pathogenic variants identified in *FLT4/VEGFR3* and, in one patient, a pathogenic variant in the *GJC2* gene. A *GJC2* mutation in a patient presenting with lymphoedema at birth is unusual but shows the variability of the phenotype.

Many of the conditions listed under the other categories in the classification algorithm may initially present with congenital lymphoedema but systemic involvement, progressive overgrowth or vascular malformation may present later and are so reclassified. Likewise, some syndromic forms may present with congenital lymphoedema before any other manifestations, making diagnosis difficult at times. Thus, the diagnosis of ‘isolated’ congenital primary lymphoedema may be difficult in a neonate presenting with pedal oedema. Therefore, a molecular diagnosis in the neonatal period is clinically very useful in the management of these patients.

### Late-onset lymphoedema (purple)

‘Late-onset’ lymphoedema is defined as presenting after the first year of life. Swelling can range from being unilateral, bilateral or can involve all four limbs and can present from early childhood up to adulthood (figures 1 and 2E). Some may present with unilateral swelling, but the contralateral limb may become involved later or show abnormalities on lymphoscintigram even when clinically uninvolved. The phenotypes also range from mild to severe. There are currently five genes known to be associated with late-onset lymphoedema: *FOXC2* (figure 2F),^27^ *GJC2*,^28 29^ *GATA2* (figure 2G),^30^ *HGF* 31 and *CELSR1* 32 (table 1). For many patients the molecular cause remains elusive, particularly in those patients with Meige disease and late-onset (usually pubertal) unilateral lower limb lymphoedema.

Late-onset primary lymphoedema accounted for 37% (n=85) in 2016 (figure 2, pie chart) comparable to the 36% reported in 2010.^8^ This category has a low number of molecular diagnoses (n=12; 14%) as there are currently no causative genes for Meige disease, which made up 36% (n=31) of patients in this category.

## Discussion

This review presents an updated St George’s classification algorithm of primary lymphatic anomalies and brings it in-line with the ISSVA classification for vascular anomalies. It cites eight new causative genes since the last publication and highlights the areas where the genetic basis is still not known. This rapidly evolving field demonstrates that primary lymphoedema and vascular malformations are highly heterogenous.

The audit reports an overall successful molecular diagnosis in 26% of patients seen in the clinic, but 41% of those patients selected for molecular testing. This is a considerable improvement on the rate of a molecular diagnosis since the algorithm was first published in 2010. Only two causal genes were known at that time. We can conclude from the audit that the algorithm works well in targeting mutation testing. Furthermore, use of the algorithm has led to the discovery of a number of causal genes. While it could be argued that the introduction of the lymphoedema gene panel obviates any need for targeted gene tests, we believe that matching a phenotype to a likely gene reduces wasteful testing and helps enormously in the interpretation of variants of unknown significance, which are becoming an increasing problem in the era of next-generation sequencing.

Although providing a molecular diagnosis in one-quarter of all the patients with primary lymphoedema represents a considerable improvement from when the algorithm was last reviewed, the molecular diagnosis is still not identified in the majority of patients seen in the St George’s Clinic. In the diagnostic setting, the introduction of next-generation sequencing with a targeted (virtual) ‘lymphoedema gene panel’ may improve the diagnostic rate and broaden the phenotypic spectrum of many of the known genetic disorders. Understanding of the natural history of the disorder will enable appropriate surveillance of, for example, leukaemia in Emberger syndrome (*GATA2*), and allow investigations for known associated problems, for example, congenital heart disease in patients with lymphoedema distichiasis syndrome (*FOXC2*). Prenatal diagnosis for the more serious conditions also becomes possible. Knowledge of causal genes, and mechanisms of pathophysiology, provide an opportunity for new, improved treatments (personalised medicine) (eg, mammalian target of rapamycin inhibitors for progressive overgrowth disorders).

In conclusion, the St George’s classification algorithm for primary lymphatic anomalies has been further refined. With this review, we have provided insight into the most recently discovered genotypes and how this algorithm can be used in the clinic to guide management of patients with primary lymphoedema.
