# Supplementary material for: Update and audit of the St George’s classification algorithm of primary lymphatic anomalies: a clinical and molecular approach to diagnosis
Source: J Med Genet. 2020 May 14;57(10):653–9. doi: 10.1136/jmedgenet-2019-106084 (PMC7525776; doi:10.1136/jmedgenet-2019-106084)
Supplement: Supplementary data [file jmedgenet-2019-106084supp001.pdf]

Supplementary Section

Supplementary Section

RECENTLY DESCRIBED NEW GENETIC ENTITIES

EPHB4 – Autosomal dominant lymphatic-related hydrops fetalis (MIM: 617300)

Heterozygous *EPHB4* mutations were identified following WES in two families, who had a significant family history of antenatal, non-immune fetal hydrops and atrial septal defects consistent with autosomal dominant inheritance.[1] This phenotype is characterised by fetal hydrops or antenatal pleural effusions (or postnatal chylothoraces), which vary in severity and, in several cases, have led to fetal demise. The hydrops may resolve in the neonatal period, but patients may develop peripheral oedema later in life (Supplemental Figure 1A), which mainly affects the legs. A high incidence of early onset and severe varicose veins was observed (Supplemental Figure 1B).

Recently, a pathogenic heterozygous variant in *EPHB4* was identified in a 4-generation family with a central conducting lymphatic anomaly.[2] This family also had a child presenting with fetal hydrops, and probably represents a variation of the same condition. Venous insufficiency was also very prevalent in this family.

Interestingly, pathogenic *EPHB4* variants have also been identified in families and singletons with vein of Galen aneurysmal malformations [3 4] and capillary malformation-arteriovenous malformation [5 6], the latter showing phenotypic overlap with hereditary haemorrhagic telangiectasia.

PIEZO1 - Generalized lymphatic dysplasia of Fotiou (MIM:616843)

PIEZO1-associated hereditary lymphoedema is an autosomal recessive generalized lymphatic dysplasia, often presenting prenatally as fetal hydrops.[7 8] Biallelic loss-of-function mutations in the *PIEZO1* gene cause a variable phenotype. The oedema can be severe, in

Supplementary Section

some cases leading to perinatal death. However, it may resolve in infancy and may re-present as peripheral lymphoedema during childhood. The swelling frequently involves the lower limbs, genitalia and face (Supplemental Figure 1C, D). There is often swelling of the upper limbs, but this is not as marked (Supplemental Figure 1E). There may be chylothoraces, pericardial effusions and, rarely, intestinal lymphangiectasia.

Heterozygous gain-of-function mutations in *PIEZO1* cause autosomal dominant dehydrated hereditary stomatocytosis (MIM: 194380), a relatively mild anaemia, which may also present with perinatal oedema/fetal hydrops (not due to anaemia).[9 10]

ADAMTS3 - Hennekam lymphangiectasia-lymphoedema syndrome 3 (MIM:618154)

ADAMTS3, together with *CCBE1*, is essential for the proteolytic activation of pro-VEGFC, the ligand for VEGFR3.[11-13] One family, including two affected children, presenting with a severe generalised lymphatic dysplasia (Hennekam lymphangiectasia-lymphoedema syndrome type 3) has been published.[14] The features include: antenatal polyhydramnios, hydroceles, congenital lymphoedema of the lower limbs and genitalia, intestinal lymphangiectasia with a protein losing enteropathy, and distinctive facial features probably secondary to facial oedema. Whole exome sequencing (WES) in the family identified compound heterozygous variants in *ADAMTS3*. Functional analysis of the variants (c.503T>C and c.872T>C) confirmed them to be highly damaging.

FAT4 - Hennekam lymphangiectasia–lymphoedema syndrome 2 (MIM: 616006)

Alders and colleagues described biallelic mutations in *FAT4* in nine patients from five families, who all presented with lymphoedema of the extremities, presenting at birth or in childhood.[15] Seven of the nine had intestinal lymphangiectasia with or without other systemic involvement. Mutations in *FAT4* have previously been found to cause Van Maldergem syndrome 2 (MIM: 615546).[16 17] Many features of the two allelic conditions

Supplementary Section

overlap; including facial dysmorphism (hypertelorism, epicanthus and a flat nasal bridge), impaired cognition, small ears with thick helices and irregular dentition. In addition, Van Maldergem syndrome is associated with neonatal hypotonia and feeding problems, hearing loss, tracheal anomalies, and osteopenia[18] and lymphoedema has been reported in one patient (Supplemental Figure 1F).[19]

FBXL7 biallelic mutations associated with Hennekam syndrome

A recent publication describes a homozygous single-exon deletion affecting *FBXL7* in a patient presenting with Hennekam syndrome. Previous studies in Drosophila had indicated that Fbxl7 interacts with Fat, of which human FAT4 is an ortholog. The patient presented shortly after birth with facial and scrotal oedema, which resolved. However, at 3 months of age, he developed persistent bilateral lower limb oedema. Clinically, he had intestinal lymphangiectasia, although this was not confirmed by endoscopic examination. Dysmorphic facies and camptodactyly, in keeping with those seen in Hennekam lymphangiectasia-lymphoedema type 2 (*FAT4*-associated), are described but photographs of the patient are not included.[20]

Mutations in any of the genes in the "Lymphoedema with Systemic Involvement" (pink) category may initially present with congenital lymphoedema, as many of the 'systemic' symptoms may not be present at birth but develop later in childhood. Therefore, this group of genes/conditions (*ADAMTS3, CCBE1, EPHB4, FAT4, PIEZO1* and *SOX18*) should be considered in any infant presenting with congenital lymphoedema, particularly if the swelling is not confined to the lower limbs, and if the genital region is swollen at birth (Figure 1). This category should be considered in any patient presenting prenatally with fetal hydrops.

Supplementary Section

CELSR1 associated with hereditary late onset primary lymphoedema

Three recent publications present the identification of truncating variants of *CELSR1* in families with an autosomal dominant, non-syndromic lymphoedema of the lower limbs presenting in childhood and predominantly affecting females.[21-23] Imaging showed extensive dermal backflow with tortuous lymphatic vessels.[22] CELSR1 is an atypical cadherin involved in planar cell polarity. Previous work had demonstrated a critical role of this gene in intraluminal valve formation in murine lymphatic vessels.[24]

PIK3CA Related Overgrowth Spectrum (PROS)

The PIK3CA-Related Overgrowth Spectrum (PROS) includes a range of mosaic conditions caused by postzygotic, gain-of-function mutations in *PIK3CA*.[25] The phenotypic spectrum of PROS includes disorders which have overlapping clinical features: Fibroadipose hyperplasia[26]; isolated lymphatic malformation;[27] CLOVES (congenital lipomatous overgrowth, vascular malformations, epidermal naevi, scoliosis/skeletal and spinal)[28] and megalencephaly-capillary malformations (MCAP) and Klippel-Trenaunay syndrome.[25 29] Lymphatic manifestations may, or may not, be present.

The PROS spectrum is characterised by asymmetrical and disproportionate congenital (or early childhood) onset of segmental overgrowth, which may be progressive. Additional features include epidermal naevi, vascular malformations, macrodactyly and macrocephaly. The severity of PROS varies; the overgrowth can be mild with very little progression (e.g. macrodactyly of one digit) or it can be extreme (affecting a range of tissues including adipose, muscular and skeletal) (Supplemental Figure 1G).[26]

Pharmacologic therapy with mTOR inhibitors for patients with progressive overgrowth has been introduced, with variable success in halting the progressive overgrowth.[30-32] Since PROS was originally described, Klippel-Trenaunay syndrome (KTS) has been included

Supplementary Section

within the spectrum, as *PIK3CA* mutations have been identified in some patients with KTS.[33] However, Proteus syndrome, another mosaic phenotype, remains separate due to the presence of a cerebriform connective tissue naevi (usually on the plantar surface) and distinct causal postzygotic, gain-of-function mutations in the *AKT1* gene.

Mosaic RASopathies

Recent publications describe patients with postzygotic mosaic mutations in the RASopathy genes resulting in high-flow arteriovenous malformations and low-flow vascular malformations with a somatic mosaic distribution.[30] One of these patients (Case 15), was seen in our clinic with congenital, unilateral lymphoedema of the left lower limb (Figure 2C). He required epiphyseal fusion to prevent increasing limb length discrepancy due to segmental overgrowth of the left leg. There was an extensive vascular malformation of the left leg and venous duplex confirmed venous incompetence. Lymphoscintigraphy also confirmed lymph drainage abnormalities. His presentation was consistent with a diagnosis of Klippel-Trenaunay syndrome. A postzygotic mutation, resulting in somatic mosaicism, was identified in the *KRAS* gene with a variant allele frequency (VAF) of only 2% identified in DNA extracted from a skin biopsy from the affected leg. This KRAS variant, c.35G>A;p.(Gly12Asp) has previously only been seen in association with cancer but never seen as a germline mutation in Noonan syndrome or Cardiofaciocutaneous syndrome.[34]

We have seen one further patient with an extensive vascular malformation of his right lower limb associated with lymphoedema. A pathogenic *MAP2K1* variant, c.360G>T;p.(Glu120Asp), was identified with a variant allele frequency (VAF) of 49% in DNA extracted from skin fibroblasts in the affected leg. This variant was not seen in the DNA extracted from blood lymphocytes.

Supplementary Section

Interestingly, different germline mutations in the same genes cause Noonan syndrome and Cardiofaciocutaneous syndrome, both of which are also known to be complicated by lymphatic abnormalities.[35] Therefore, a mosaic RASopathy should be suspected in any patient with segmental overgrowth, vascular malformations with or without lymphoedema, in whom no mutation in *PIK3CA* has been identified.

Supplementary Section

Supplementary Figure 1: Clinical photos of patients with mutations in some of the new causal genes.

(A-B) Mutations in *EPHB4* cause autosomal dominant lymphatic-related fetal hydrops. In adults it can lead to mild facial oedema (A) and extensive varicose veins (arrow) (B). (C-E) Mutations in *PIEZO1* cause a type of generalised lymphatic dysplasia. Patients can present with facial oedema (C) and four limb lymphoedema (D, E). (F) Typical facies of Van Maldergem syndrome caused by mutations in *FAT4*. (G) PROS-overgrowth of left leg with vascular malformation caused by a postzygotic mutation in *PIK3CA*.

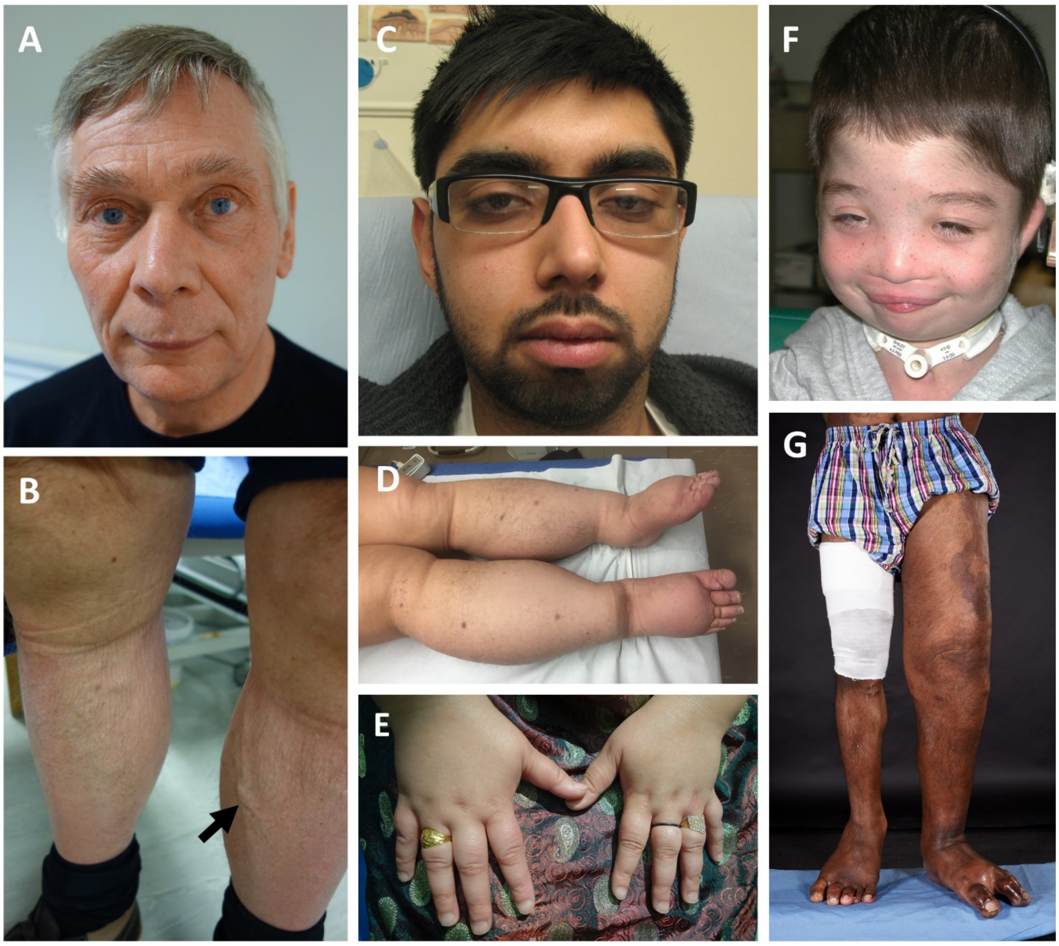

Supplementary Section

GLOSSARY OF TERMS

In order to maximise the utility of the St. George's classification algorithm, it is helpful to define some of the terms used:

Lymphoedema: swelling of an extremity due to lymphatic dysfunction (i.e. not oedema from, for example, heart failure or an allergic reaction). Involvement of one or more extremities is peripheral lymphoedema.

Primary lymphoedema: Lymphoedema due to a developmental fault in the structure or function of the lymph conducting pathways and presumed to be genetic in origin. Thus, it is not **secondary** to an identified cause (e.g. cancer or infection).

Lymphatic malformation: These are overt structural defects of the lymph conducting pathways, which may include **truncal malformations** (if interfering with lymph drainage and cause lymphoedema) or **non-truncal malformations, (**isolated anomalies with no connection to main lymph drainage pathways and do not cause lymphoedema).

Generalised Lymphatic Dysplasia (dys = bad; plasis = formation) is used to describe the abnormal growth/development of the lymphatic system and is a structural or functional abnormality.

Systemic involvement or internal/visceral lymphatic dysfunction: Abnormal lymphatic function causing internal swelling e.g. chylothoraces, chylopericardium, chylous ascites, intestinal or pulmonary lymphangiectasia or non-immune fetal hydrops.

Central conducting lymphatic anomalies (CCLA) is a term that is used to describe dysfunction or obstruction of the lymph conducting channels within the thorax or abdomen as seen on imaging such as contrast-enhanced MR Lymphangiography.[36] This may present as chylothoraces, chylopericardium, ascites and chylous reflux with leaking of lymphatic fluid,

Supplementary Section

often into the genital area. Causes of CCLA include Noonan syndrome (under 'Syndromic lymphoedema' [blue section]),[37] *EPHB4*-associated disorders[2] (under 'Generalised lymphoedema with systemic involvement' [pink section]). It is therefore a highly heterogenous, descriptive term rather than a specific entity.

Generalised lymphatic anomaly (GLA) is a term that is easily confused with generalised lymphatic dysplasia (see describtion above). However, generalised lymphatic anomaly (GLA), also called lymphangiomatosis, is a rare condition involving the abnormal overgrowth of lymphatic vessels (small and large cystic lymphangiomas) in the lungs, pleura, bones and soft tissue. GLAs may be congenital or acquired. The lymphangiomas may initially be proliferative, but then stabilise over time. In others, they may follow a progressive course and result in life-threatening complications, pain and functional disability. We have included Gorham-Stout disease (GSD) in this category, a condition involving abnormal growth of lymphatic vessels that affects bone.[38] The bony destruction (osteolysis) is progressive in GSD, hence the name "vanishing bone disease", whereas bony lytic lesions in GLA are less aggressive. Causal mutations have not yet been discovered for GLA or GSD, but therapeutic trials of sirolimus have been successful in some patients.[39]

Supplementary Section

References

1. Martin-Almedina S, Martinez-Corral I, Holdhus R, Vicente A, Fotiou E, Lin S, Petersen K, Simpson MA, Hoischen A, Gilissen C, Jeffery H, Atton G, Karapouliou C, Brice G, Gordon K, Wiseman JW, Wedin M, Rockson SG, Jeffery S, Mortimer PS, Snyder MP, Berland S, Mansour S, Makinen T and Ostergaard P. EPHB4 kinase-inactivating mutations cause autosomal dominant lymphatic-related hydrops fetalis. *Journal of Clinical Investigation*. 2016;126:3080-3088.

2. Li D, Wenger TL, Seiler C, March ME, Gutierrez-Uzquiza A, Kao C, Bhoj E, Tian L, Rosenbach M, Liu Y, Robinson N, Behr M, Chiavacci R, Hou C, Wang T, Bakay M, Pellegrino da Silva R, Perkins JA, Sleiman P, Levine MA, Hicks PJ, Itkin M, Dori Y and Hakonarson H. Pathogenic variant in EPHB4 results in central conducting lymphatic anomaly. *Hum Mol Genet*. 2018;27:3233-3245.

3. Vivanti A, Ozanne A, Grondin C, Saliou G, Quevarec L, Maurey H, Aubourg P, Benachi A, Gut M, Gut I, Martinovic J, Senat MV, Tawk M and Melki J. Loss of function mutations in EPHB4 are responsible for vein of Galen aneurysmal malformation. *Brain*. 2018;141:979-988.

4. Duran D, Zeng X, Jin SC, Choi J, Nelson-Williams C, Yatsula B, Gaillard J, Furey CG, Lu Q, Timberlake AT, Dong W, Sorscher MA, Loring E, Klein J, Allocco A, Hunt A, Conine S, Karimy JK, Youngblood MW, Zhang J, DiLuna ML, Matouk CC, Mane S, Tikhonova IR, Castaldi C, Lopez-Giraldez F, Knight J, Haider S, Soban M, Alper SL, Komiyama M, Ducruet AF, Zabramski JM, Dardik A, Walcott BP, Stapleton CJ, Aagaard-Kienitz B, Rodesch G, Jackson E, Smith ER, Orbach DB, Berenstein A, Bilguvar K, Vikkula M, Gunel M, Lifton RP and Kahle KT. Mutations in Chromatin Modifier and Ephrin Signaling Genes in Vein of Galen Malformation. *Neuron*. 2019;101:429-443.e4.

5. Amyere M, Revencu N, Helaers R, Pairet E, Baselga E, Cordisco M, Chung W, Dubois J, Lacour JP, Martorell L, Mazereeuw-Hautier J, Pyeritz RE, Amor DJ, Bisdorff A, Blei F, Bombei H, Dompmartin A, Brooks D, Dupont J, Gonzalez-Ensenat MA, Frieden I, Gerard M, Kvarnung M, Hanson-Kahn AK, Hudgins L, Leaute-Labreze C, McCuaig C, Metry D, Parent P, Paul C, Petit F, Phan A, Quere I, Salhi A, Turner A, Vabres P, Vicente A, Wargon O, Watanabe S, Weibel L, Wilson A, Willing M, Mulliken JB, Boon LM and Vikkula M. Germline Loss-of-Function Mutations in EPHB4 Cause a Second Form of Capillary Malformation-Arteriovenous Malformation (CM-AVM2) Deregulating RAS-MAPK Signaling. *Circulation*. 2017;136:1037-1048.

6. Yu J, Streicher JL, Medne L, Krantz ID and Yan AC. EPHB4 Mutation Implicated in Capillary Malformation-Arteriovenous Malformation Syndrome: A Case Report. *Pediatr Dermatol*. 2017;34:e227-e230.

7. Fotiou E, Martin-Almedina S, Simpson MA, Lin S, Gordon K, Brice G, Atton G, Jeffery I, Rees DC, Mignot C, Vogt J, Homfray T, Snyder MP, Rockson SG, Jeffery S, Mortimer PS, Mansour S and Ostergaard P. Novel mutations in PIEZO1 cause an autosomal recessive generalized lymphatic dysplasia with non-immune hydrops fetalis. *Nature Communications*. 2015;6:8085.

8. Lukacs V, Mathur J, Mao R, Bayrak-Toydemir P, Procter M, Cahalan SM, Kim HJ, Bandell M, Longo N, Day RW, Stevenson DA, Patapoutian A and Krock BL. Impaired PIEZO1 function in patients with a novel autosomal recessive congenital lymphatic dysplasia. *Nat Commun*. 2015;6:8329.

9. Albuisson J, Murthy SE, Bandell M, Coste B, Louis-dit-Picard H, Mathur J, Feneant-Thibault M, Tertian G, de Jaureguiberry J-P, Syfuss P-Y, Cahalan S, Garcon L, Toutain F, Rohrlich PS, Delaunay J, Picard V, Jeunemaitre X and Patapoutian A. Dehydrated hereditary stomatocytosis linked to gain-of-function mutations in mechanically activated PIEZO1 ion channels. *Nature Communications*. 2013;4:1884.

Supplementary Section

10. Martin-Almedina S, Mansour S and Ostergaard P. Human phenotypes caused by PIEZO1 mutations; one gene, two overlapping phenotypes? *J Physiol*. 2018;596:985-992.
11. Bui HM, Enis D, Robciuc MR, Nurmi HJ, Cohen J, Chen M, Yang Y, Dhillon V, Johnson K, Zhang H, Kirkpatrick R, Traxler E, Anisimov A, Alitalo K and Kahn ML. Proteolytic activation defines distinct lymphangiogenic mechanisms for VEGFC and VEGFD. *J Clin Invest*. 2016;126:2167-80.
12. Jha SK, Rauniyar K, Karpanen T, Leppanen VM, Brouillard P, Vikkula M, Alitalo K and Jeltsch M. Efficient activation of the lymphangiogenic growth factor VEGF-C requires the C-terminal domain of VEGF-C and the N-terminal domain of CCBE1. *Sci Rep*. 2017;7:4916.
13. Jeltsch M, Jha SK, Tvorogov D, Anisimov A, Leppanen V-M, Holopainen T, Kivela R, Ortega S, Karpanen T and Alitalo K. CCBE1 Enhances Lymphangiogenesis via A Disintegrin and Metalloprotease With Thrombospondin Motifs-3-Mediated Vascular Endothelial Growth Factor-C Activation. *Circulation*. 2014;129:1962-1971.
14. Brouillard P, Dupont L, Helaers R, Coulie R, Tiller GE, Peeden J, Colige A and Vikkula M. Loss of ADAMTS3 activity causes Hennekam lymphangiectasia-lymphedema syndrome 3. *Hum Mol Genet*. 2017;26:4095-4104.
15. Alders M, Al-Gazali L, Cordeiro I, Dallapiccola B, Garavelli L, Tuysuz B, Salehi F, Haagmans MA, Mook OR, Majoie CB, Mannens MM and Hennekam RC. Hennekam syndrome can be caused by FAT4 mutations and be allelic to Van Maldergem syndrome. *Human Genetics*. 2014;133:1161-1167.
16. Cappello S, Gray MJ, Badouel C, Lange S, Einsiedler M, Srour M, Chitayat D, Hamdan FF, Jenkins ZA, Morgan T, Preitner N, Uster T, Thomas J, Shannon P, Morrison V, Di Donato N, Van Maldergem L, Neuhann T, Newbury-Ecob R, Swinkells M, Terhal P, Wilson LC, Zwijnenburg PJ, Sutherland-Smith AJ, Black MA, Markie D, Michaud JL, Simpson MA, Mansour S, McNeill H, Gotz M and Robertson SP. Mutations in genes encoding the cadherin receptor-ligand pair DCHS1 and FAT4 disrupt cerebral cortical development. *Nat Genet*. 2013;45:1300-8.
17. Van Maldergem L, Wetzburger C, Verloes A, Fourneau C and Gillerot Y. Mental retardation with blepharo-naso-facial abnormalities and hand malformations: a new syndrome? *Clin Genet*. 1992;41:22-4.
18. Ivanovski I, Akbaroghli S, Pollazzon M, Gelmini C, Caraffi SG, Mansouri M, Chavoshzadeh Z, Rosato S, Polizzi V, Gargano G, Alders M, Garavelli L and Hennekam RC. Van Maldergem syndrome and Hennekam syndrome: Further delineation of allelic phenotypes. *Am J Med Genet A*. 2018;176:1166-1174.
19. Mansour S, Swinkels M, Terhal PA, Wilson LC, Rich P, Van Maldergem L, Zwijnenburg PJ, Hall CM, Robertson SP and Newbury-Ecob R. Van Maldergem syndrome: further characterisation and evidence for neuronal migration abnormalities and autosomal recessive inheritance. *Eur J Hum Genet*. 2012;20:1024-31.
20. Boone PM, Paterson S, Mohajeri K, Zhu W, Genetti CA, Tai DJC, Nori N, Agrawal PB, Bacino CA, Bi W, Talkowski ME, Hogan BM and Rodan LH. Biallelic mutation of FBXL7 suggests a novel form of Hennekam syndrome. *Am J Med Genet A*. 2020;182:189-194.
21. Erickson RP, Lai LW, Mustacich DJ, Bernas MJ, Kuo PH and Witte MH. Sex-limited penetrance of lymphedema to females with CELSR1 haploinsufficiency: A second family. *Clin Genet*. 2019;96:478-482.
22. Gonzalez-Garay ML, Aldrich MB, Rasmussen JC, Guilliod R, Lapinski PE, King PD and Sevick-Muraca EM. A novel mutation in CELSR1 is associated with hereditary lymphedema *Vasc Cell*; 2016;8:1.

Supplementary Section

23. Maltese PE, Michelini S, Ricci M, Maitz S, Fiorentino A, Serrani R, Lazzerotti A, Bruson A, Paolacci S, Benedetti S and Bertelli M. Increasing evidence of hereditary lymphedema caused by CELSR1 loss-of-function variants. *Am J Med Genet A*. 2019;179:1718-1724.
24. Tatin F, Taddei A, Weston A, Fuchs E, Devenport D, Tissir F and Makinen T. Planar cell polarity protein Celsr1 regulates endothelial adherens junctions and directed cell rearrangements during valve morphogenesis. *Dev Cell*. 2013;26:31-44.
25. Keppler-Noreuil KM, Rios JJ, Parker VE, Semple RK, Lindhurst MJ, Sapp JC, Alomari A, Ezaki M, Dobyns W and Biesecker LG. PIK3CA-related overgrowth spectrum (PROS): diagnostic and testing eligibility criteria, differential diagnosis, and evaluation. *Am J Med Genet A*. 2015;167A:287-95.
26. Lindhurst MJ, Parker VER, Payne F, Sapp JC, Rudge S, Harris J, Witkowski AM, Zhang Q, Groeneveld MP, Scott CE, Daly A, Huson SM, Tosi LL, Cunningham ML, Darling TN, Geer J, Gucev Z, Sutton VR, Tziotzios C, Dixon AK, Helliwell T, O'Rahilly S, Savage DB, Wakelam MJO, Barroso I, Biesecker LG and Semple RK. Mosaic overgrowth with fibroadipose hyperplasia is caused by somatic activating mutations in PIK3CA. *Nature Genetics*. 2012;44:928-933.
27. Luks VL, Kamitaki N, Vivero MP, Uller W, Rab R, Bovee JV, Rialon KL, Guevara CJ, Alomari AI, Greene AK, Fishman SJ, Kozakewich HP, Maclellan RA, Mulliken JB, Rahbar R, Spencer SA, Trenor CC, 3rd, Upton J, Zurakowski D, Perkins JA, Kirsh A, Bennett JT, Dobyns WB, Kurek KC, Warman ML, McCarroll SA and Murillo R. Lymphatic and other vascular malformative/overgrowth disorders are caused by somatic mutations in PIK3CA. *J Pediatr*. 2015;166:1048-54.e1-5.
28. Kurek K, Luks V, Ayturk U, Alomari A, Fishman S, Spencer S, Mulliken J, Bowen M, Yamamoto G, Kozakewich H and Warman M. Somatic Mosaic Activating Mutations in PIK3CA Cause CLOVES Syndrome *Am J Hum Genet*; 2012(90): 1108-15.
29. Keppler-Noreuil KM, Sapp JC, Lindhurst MJ, Parker VE, Blumhorst C, Darling T, Tosi LL, Huson SM, Whitehouse RW, Jakkula E, Grant I, Balasubramanian M, Chandler KE, Fraser JL, Gucev Z, Crow YJ, Brennan LM, Clark R, Sellars EA, Pena LD, Krishnamurty V, Shuen A, Braverman N, Cunningham ML, Sutton VR, Tasic V, Graham JM, Jr., Geer J, Jr., Henderson A, Semple RK and Biesecker LG. Clinical delineation and natural history of the PIK3CA-related overgrowth spectrum. *Am J Med Genet A*. 2014;164a:1713-33.
30. Al-Olabi L, Polubothu S, Dowsett K, Andrews KA, Stadnik P, Joseph AP, Knox R, Pittman A, Clark G, Baird W, Bulstrode N, Glover M, Gordon K, Hargrave D, Huson SM, Jacques TS, James G, Kondolf H, Kangesu L, Keppler-Noreuil KM, Khan A, Lindhurst MJ, Lipson M, Mansour S, O'Hara J, Mahon C, Mosica A, Moss C, Murthy A, Ong J, Parker VE, Riviere JB, Sapp JC, Sebire NJ, Shah R, Sivakumar B, Thomas A, Virasami A, Waelchli R, Zeng Z, Biesecker LG, Barnacle A, Topf M, Semple RK, Patton EE and Kinsler VA. Mosaic RAS/MAPK variants cause sporadic vascular malformations which respond to targeted therapy. *J Clin Invest*. 2018;128:1496-1508.
31. Keppler-Noreuil KM, Parker VE, Darling TN and Martinez-Agosto JA. Somatic overgrowth disorders of the PI3K/AKT/mTOR pathway & therapeutic strategies. *Am J Med Genet C Semin Med Genet*. 2016;172:402-421.
32. Parker VE, Knox RG, Zhang Q, Wakelam MJ and Semple RK. Phosphoinositide 3-kinase-related overgrowth: cellular phenotype and future therapeutic options. *Lancet*. 2015;385 Suppl 1:S77.
33. Vahidnezhad H, Youssefian L and Uitto J. Klippel-Trenaunay syndrome belongs to the PIK3CA-related overgrowth spectrum (PROS). *Exp Dermatol*. 2016;25:17-9.

Supplementary Section

34. Chen CC, Er TK, Liu YY, Hwang JK, Barrio MJ, Rodrigo M, Garcia-Toro E and Herreros-Villanueva M. Computational analysis of KRAS mutations: implications for different effects on the KRAS p.G12D and p.G13D mutations. *PLoS One*. 2013;8:e55793.
35. Joyce S, Gordon K, Brice G, Ostergaard P, Nagaraja R, Short J, Moore S, Mortimer P and Mansour S. The lymphatic phenotype in Noonan and Cardiofaciocutaneous syndrome. *European Journal of Human Genetics*. 2016;24:690-696.
36. Chavhan GB, Amaral JG, Temple M and Itkin M. MR Lymphangiography in Children: Technique and Potential Applications. *Radiographics*. 2017;37:1775-1790.
37. Biko DM, Reisen B, Otero HJ, Ravishankar C, Victoria T, Glatz AC, Rome JJ and Dori Y. Imaging of central lymphatic abnormalities in Noonan syndrome. *Pediatr Radiol*. 2019;49:586-592.
38. Rossi M, Buonuomo PS, Battafarano G, Conforti A, Mariani E, Algeri M, Pelle S, D'Agostini M, Macchiaiolo M, De Vito R, Gonfiantini MV, Jenkner A, Rana I, Bartuli A and Del Fattore A. Dissecting the mechanisms of bone loss in Gorham-Stout disease. *Bone*. 2020;130:115068.
39. Ricci KW, Hammill AM, Mobberley-Schuman P, Nelson SC, Blatt J, Bender JLG, McCuaig CC, Synakiewicz A, Frieden IJ and Adams DM. Efficacy of systemic sirolimus in the treatment of generalized lymphatic anomaly and Gorham-Stout disease. *Pediatr Blood Cancer*. 2019;66:e27614.
